# Understanding the Challenges of Improving Sanitation and Hygiene Outcomes in a Community Based Intervention: A Cross-Sectional Study in Rural Tanzania

**DOI:** 10.3390/ijerph14060602

**Published:** 2017-06-05

**Authors:** Joseph Kihika Kamara, Moses Galukande, Florence Maeda, Sam Luboga, Andre M. N. Renzaho

**Affiliations:** 1 School of Social Sciences and Psychology, Western Sydney University, Sydney, Locked Bag 1797, Penrith NSW 2751, Australia; 2 World Vision International, Southern Africa Regional Office, Mbabane H100, Swaziland; 3 Makerere University College of Health Sciences, P.O. Box 7072, Kampala, Uganda; mosesg@img.co.ug; 4 World Vision Tanzania, Nzega, P.O. Box 614, Tabora, Tanzania; florence_maeda@wvi.org; 5 Sustainable Leadership Institute, P.O. Box 1532, Kampala, Uganda; lubogasam@gmail.com; 6 Humanitarian and Development Research Initiative, School of Social Sciences and Psychology, Western Sydney University, Sydney, Locked Bag 1797, Penrith NSW 2751, Australia; Andre.Renzaho@westernsydney.edu.au

**Keywords:** water, sanitation, hygiene, diarrhea, rural communities

## Abstract

Good sanitation and clean water are basic human rights yet they remain elusive to many rural communities in Sub-Saharan Africa (SSA). We carried out a cross sectional study to examine the impact of a four-year intervention aimed at improving access to water and sanitation and reducing waterborne disease, especially diarrhea in children under five years old. The study was carried out in April and May 2015 in Busangi, Chela and Ntobo wards of Kahama District of Tanzania. The interventions included education campaigns and improved water supply, and sanitation. The percentage of households (HHs) with access to water within 30 min increased from 19.2 to 48.9 and 17.6 to 27.3 in the wet and dry seasons, respectively. The percentage of HHs with hand washing facilities at the latrine increased from 0% to 13.2%. However, the incidence of diarrhea among children under five years increased over the intervention period, RR 2.91 95% CI 2.71–3.11, *p* < 0.0001. Availability of water alone may not influence the incidence of waterborne diseases. Factors such as water storage and usage, safe excreta disposal and other hygiene practices are critical for interventions negating the spread of water borne diseases. A model that articulates the extent to which these factors are helpful for such interventions should be explored.

## 1. Introduction

Sub-Saharan Africa has very low water and basic sanitation coverage despite good sanitation and safe drinking water being fundamental for wellbeing and basic human rights [1,2]. Consequently, water, sanitation and hygiene have been the focus of development goals. The Millennium Development Goal (MDG) seven sought to halve the proportion of the population without sustainable access to safe drinking water and basic sanitation. However, sanitation remains poorly resourced and understood, resulting in limited progress [1,3]. The 2015 MDG final report outlines that, although five developing regions met the access to drinking water target, Sub-Saharan Africa (SSA) fell short of meeting the target. However, the region had a 20% percentage point increase in the use of improved sources of drinking water during the MDG monitoring period [4]. In SSA, the proportion of population using improved drinking water sources increased from 48% in 1990 to 68% in 2015 (vs. 89% in the developing region and 91% in the world). SSA remains the region with the highest number of people without access to improved sources of drinking water in the world. Of the estimated 663 million people worldwide still using unimproved drinking water sources (e.g., unprotected wells and springs and surface water), nearly half of them (319 million people) live in sub-Saharan Africa [4]. Nonetheless, the proportion of people using improved sanitation facilities increased from 24% in 1990 to 30% in 2015 [4].

Sanitation remains one of Africa’s major public health challenges. On average, half of the population in Sub-Saharan Africa does not use appropriate facilities. Poor sanitation causes millions of people worldwide to contract fecal borne illnesses, the most common being diarrhea [5]. However, there are regional variations across the continent, with people living in rural areas less likely to have access to improved water and sanitation facilities. To eliminate inequalities in access to water and sanitation, interventions that target the most vulnerable populations with improved water sources are required. However, improved water sources are not always free of contamination; they require constant monitoring for quality. Some studies have identified community participation, cleaning of transportation and storage containers as well as boiling and chlorination that can be combined with improved water sources to enhance health outcomes [6,7,8]. 

In Tanzania, only 55% and 15.4% of the country’s population has access to safe water and sanitation, respectively, and most of these are in urban areas [9]. Rural areas have substandard water systems with 46% and a dismal 8% of the population with access to improved water and sanitation, respectively [1]. Tanzania has appropriate water, sanitation and hygiene governance policies, and has made efforts to improve safe water, sanitation and hygiene. However, the country only made limited progress toward the attainment of the MDG 7c; to halve the population without sustainable access to safe drinking water and basic sanitation. Between 1990 and 2015, a dismal 12% of the population gained access to improved water and sanitation [1]. Over 18,500 children less than five years old die annually due to diarrhea resulting from contaminated water and hygiene practices [10,11]. Additionally, 12.1% of total deaths in the country were related to poor water, sanitation and hygiene [12]. 

In Tanzania, adequate water coverage is when a HH is connected to a water grid or has access to a public water kiosk or a borehole that can be accessed within 30 min [13]. However, HH water connection remains a domain of the urban elite high and middle classes. This group of people lives in easy to reach areas serviced by grids, can afford the service and are adequately empowered to advocate for access to services [14,15]. The majority of the population in rural areas receive their water from unimproved sources without sufficient quality monitoring capacity. Furthermore, the population receiving such water is not recognized by the government as having adequate access. Notwithstanding the water access, improved sanitation and hygiene is defined as “*the percentage of population using “improved” sanitation, meaning facilities that ensure hygienic separation of human excreta from human contact such as: flush or pour-flush toilets connected to a piped sewer system/septic tank/pit latrine, a ventilated improved pit (VIP) latrine or a pit latrine with slab*” [1,16] *(*p. 9, 50*)*. Nonetheless, 17% of the rural population remain without access to a toilet or latrine, which leads to the practice of open defecation [17]. Open defecation is one of the leading causes of water contamination with fecal matter responsible for most diarrheal diseases. Diarrheal diseases like cholera have become endemic in Tanzania partly due to poor sanitation and hygiene. Evidence suggests that cholera presents a high disease burden with over 5800 cases reported in the country annually [10]. 

The glaring water, sanitation and hygiene gaps triggered World Vision Tanzania (WVT), a Non-Governmental Organization (NGO) to intervene in Busangi rural community. Despite the intervention, there was limited understanding of the water, sanitation and hygiene status in the intervention area. The primary objective of this study was to examine the impact of a four-year intervention that sought to improve access to water and sanitation, and reduce waterborne disease, especially diarrhea in children under five years old in Busangi. This study is structured in several segments. We follow up the background with information of the study setting, methods and results. Thereafter, we discuss the results, and present the limitations and the conclusion. 

### The Study Setting

**Figure 1:**
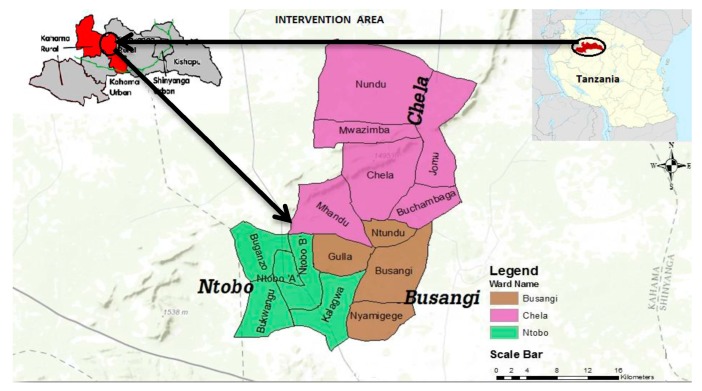
The study area. Source: [19,20,21].

The study was carried out in Busangi Area Development Program, a rural area comprised of 15 villages in Busangi, Chela and Ntobo Wards in Kahama, one of the water poor districts in Shinyanga region, Tanzania. Located in the north of Tanzania, Shinyanga borders the regions of Mwanza in the north, Tabora in the South, Gieta in the west and Simiyu in the East. The 2012 population and housing census reported that Shinyanga region has a total population of 1,534,808 with an average household of 5.9 people [18]. The same report outlined that Kahama rural district, where the study area is located, has a population of 523,802 (Male = 256,463; Female = 267,339). Of these, 11,507 live in Busangi ward, 20,760 live in Chela, and 10,089 live in Ntobo ward, the rest of the population live in other wards outside the scope of this study. The study area was conducted in 15 villages in Busangi, Chela and Ntobo wards. The total population of the study area (15 villages) is 35,978 people (adult Male = 17,566, adult Female = 18,411) with 5307 households. Figure 1 shows the study area.

## 2. Methods 

The study was a cross-sectional survey. Data collection used mixed methods. 

### 2.1. Sampling Strategy and Sample Size

The sampling unit in this study was a household, defined as a unit consisting of one or more people who live in the same dwelling and share meals. Households to be interviewed were identified using a multi-stage cluster sampling technique. In the first stage, the list of all villages in the study area and their household sizes was established with the help of local community leaders and World Vision staff. In the second stage, the number of HHs in each village to be surveyed was determined using the probability proportional to size. In the final stage, the household to be interviewed in each village was selected using systematic random sampling. The sampling interval (X) was determined by dividing the total number of households in each village with the expected sample size, and the first household to be surveyed was randomly selected by choosing a number between 1 and X. The next household to be visited was selected by adding X to the first randomly selected number, and the process continued until the required sample size for that village was obtained. In the absence of data on access to water, we assumed that 50% of the household would have access to improved drinking water sources for sample size calculation purposes. We estimate that the required sample size for the survey would be 440 participants with 80% power, a 5% significance level, and 5% margin error. Adjusting for a 10% non-response rate, our final sample size was 484 households. 

We used purposive sampling for the key informant interviews (KIIs) and focus group discussions (FGDs). Six focus group discussions (FGDs) were conducted. The FGDs included youth, men, and women with 8–12 people in each group. A total of 15 KIIs were conducted using semi-structured interview guides. The key informants were drawn from WVT staff, a government representative, community leader, religious leader, and water user representatives. In addition, we interviewed the head of the Health Center and a head teacher or school representative. The FGDs were used to understand the changes, or the lack thereof, and validating data collected by quantitative methods. Six out of 18 schools and four water sources (boreholes = 2, protected wells = 1 unprotected well = 1) were randomly selected and visited to assess the hygiene conditions, use and functionality. A pre-tested tool was used to record the findings. 

The interventions were a composite of improved water supply by sinking/constructing 17 boreholes and two protected wells. One borehole was located in a school compound, the rest were scattered across the community where the need was greatest and due to geological suitability. Six VIP latrines (3 for boys and 3 for girls) and fourteen latrines for teachers were constructed in three out of 18 schools. Additionally, six VIP latrines were constructed for the most vulnerable HHs including those with children with disabilities. The latrines for vulnerable HHs also served as demonstration units for the community. Improved sanitation and hygiene was promoted by encouraging the construction and use of permanent VIP latrines and community mobilization. In addition, educational campaigns using village health workers (VHWs), water user groups (WUGs) and primary schools environmental clubs were also promoted. The educational campaign groups were trained in participatory health and sanitation training (PHAST). PHAST is the government’s recommended approach to water, sanitation and hygiene interventions [22]. The content of the training emphasized appropriate fecal disposal and hand washing, clearing of seasonal bushes and stagnant water around households to minimize vector breeding. It also included the use of drying racks for sterilization of utensils, excavation and use of garbage disposal pits, and water treatment [23]. The content is known to influence positive health- behavior outcomes in rural communities [24,25]. The content was disseminated through village meetings, leaflets, posters, radio, and counseling during health center visits. Visits were scheduled periodically in the schools where teachers and students were trained in addition to community leaders and volunteers. Community leaders through community meetings appointed volunteer committees to oversee management of sanitation facilities and water sources. Teaching materials were adopted from the government water and sanitation (WATSAN) department. WVT, the government and the local communities played a central role in the execution of the intervention since 2011. Local rural communities were responsible for maintenance of most water facilities introduced by the intervention. Two full-time WVT staff members supervised the project supported by four part time staff. 

### 2.2. Study Variables

These included diarrhea incidence in children under 5 years (care seeking at health centers and self-reported in HH survey), water quantity in liters per person within 30 min (round trip from a water source). Other variables were the number of VIP and unimproved latrines, frequency of excreta disposal practices and frequency of exposure to sanitation, and hygiene messages. Diarrhea was defined as watery stools equal to or more than three episodes in 24 h. 

### 2.3. Document Review, Training Enumerators and Governance

We reviewed various documents including the national water policy, the WVT five-year strategy and health center reports. Other documents reviewed included the area development program assessment report, the water project design and monitoring reports, and the Tanzanian population and housing census report. All enumerators were proficient in Swahili and with good command of English. This enabled them (enumerators) to conduct interviews in Swahili and translated into English for scribing. The training covered sampling and interview techniques and ethical principles. Ethical principles emphasized included the right of a participant to refuse to respond to a question or to the entire survey. Other training content included the questionnaire overview, its pretest and refinement. The enumerator teams were briefed each morning and debriefed at the end of each day to discuss emerging challenges and solutions.

### 2.4. Ethical Considerations

Oral informed consent was obtained from all respondents in Swahili. For respondents under the age of 18, assent was obtained and consent from a parent or guardian, as was appropriate. The research authorization letter was obtained from Makerere School of Medicine Research and Ethics Committee under IRB approval code REC ref 2017070.

### 2.5. Inclusion and Exclusion Criteria 

All members of the HH who could answer the survey questions (with the help of a parent/guardian or surrogate, if applicable) were interviewed in Swahili, which was mutually comprehensible to the interviewer and the respondent. A person(s) with mental disability or a person(s) who would not answer the questions for any other reason were excluded from the survey. 

### 2.6. Data Collection, Analysis and Interpretation 

The study activities lasted 18 days, including eight fieldwork days. Twenty enumerators and three data entrants were selected and trained. A team of three enumerator supervisors, a WVT staff and the study leader monitored quality control. The quality control included daily briefing of the enumerators every morning to emphasize instructions before departure to collect data. Similarly, there was debriefing every evening to discuss and resolve the day’s data collection challenges. The supervisors and data entrants checked the survey tools for completeness and accuracy. Quantitative data were analyzed using SPSS. Qualitative data were inductively and thematically analyzed using manual coding following Braun and Clarke’s six-step approach to analyze data [26]. This involved reading transcripts and field notes followed by generating and inserting initial codes into the transcripts. Thereafter, we grouped the codes into potential themes. Subsequently, we reviewed the themes and created a thematic “map”, defined and named the themes in line with our theoretical framework before we narrated themes. 

## 3. Results

The impact in terms of diarrhea morbidity and water quantity accessed are presented in this section. We successfully surveyed 536 HHs comprising 2887 individuals in total. Table 1 shows respondents’ characteristics. 

The interventions in the intervention are illustrated in Table 2. Overall, six FGDs were conducted, two for men, two for women and two groups of the youth groups. The age range for men was 24–45, for women it was 23–65 and for the youth it was 11–18 years. The individual pupil (one on one) interviews were conducted in six schools from three wards. In total, 87 boys and girls were interviewed, the mean age was 13 years (range 10–16 years).

### 3.1. Diarrhea Morbidity and Mortality 

We quantified the correlation between diarrhea-specific morbidity and mortality based on our survey, secondary data collected from Busangi dispensary and Chela health centers. Chela health center is the referral clinic for many dispensaries. Overall, the number of children (under and above five years) with diarrhea significantly increased over the four-year period with the RR 2.91 95% CI 2.71–3.11, *p* < 0.0001. Bloody diarrhea was reported in 2.1% of cases during our study. 

### 3.2. Hand Washing

We found that only 13.2% of the HHs had hand washing facilities outside latrines. The type of hand washing facilities included improvised devices to discharge water known as tippy taps, cups and buckets, and taps connected to water tanks. Some of the washing facilities also had soap for hand washing for use after visiting the latrine. Tippy taps were observed in three out of six schools. One of the observed schools had a borehole in its compound. All schools had water storage tanks but some did not provide hand washing facilities. The water tanks in three out of the six schools were not in use due to disrepair.

### 3.3. Excreta Disposal 

The main excreta disposal facilities available to households were pit latrines. The term pit latrine included permanent VIP latrines, and temporary latrines. VIP latrines have vent pipes with fly screens attached to their top to prevent the entry and exit of flies which spread diseases. The vent pipes connect the pit to the air above the roof to ease air circulation and prevent odors. Temporary latrines are rudimentary and last for mostly one season which is about four months in a year. We observed that 252 (47%) of HHs with pit latrines had appropriate covers for the hole to prevent flies infestation. Seventy-seven (14.4%) of the HHs latrines observed had cement slabs easy to clean. Nonetheless, 45 (12.1%) of the household latrine floors were littered with feces and 92 (24.7%) had flies. However, 118 (31.8%) latrines appeared clean and safe enough for use. Our secondary data audit suggested an increase of VIP latrines from 407 to 2107 between the intervention inception and the study period. However, only 103 (27.7%) of the sampled HHs were found with commensurate shelter and door for privacy, and met the VIP criteria. 

There were some barriers to the use of latrines, especially the VIP type. Barriers included the inability of households to construct latrines, and the high cost involved. FGD data suggest that the labor costs involved in digging latrine pits range from 5000 and 8000 Tanzanian Shillings (TShs). This was equivalent to USD 3 per foot of depth. The standard pit latrines have an average depth of 9–12 feet. There are other costs involved in the construction to cover the slab, walls and roofing. Respondents highlighted the high construction cost of latrines as captured in one of the FGDs.

“There are few latrines, not every family has one, and therefore use is limited to those who have.” Women FGD. “VIP latrines are expensive to build. They cost up to 1,000,000 TShs. (USD 550) to construct one, especially if it is done to the standards recommended by World Vision.” Men FGD. 

The cost was unaffordable for most HHs who were subsistence farmers. Unless, the HHs are assisted with the cost, VIP latrines may remain out of reach for most of them. This could explain the persistent 40% open defecation captured in the HH survey, despite the education campaigns. Even though PHAST was deployed to promote sanitation and hygiene, we found less than 10% of HH with latrines equipped with hand-washing facilities including soap. We visited six primary schools to assess excreta disposal and hand washing behavior. Each latrine block consisted of six separated rooms also called stances. One school had a stance:pupil ratio close to 1:20. The largest ratio was 1:68 for boys and 1:64 for girls. Half of the pit latrines had wet floors with urine; some floors were littered with feces. Most of the latrines observed were smelly and only one school had two tippy taps, but had no soap for hand washing as presented in Table 3. 

### 3.4. Water Access 

Overall, people used all the water sources, including the unprotected wells for domestic chores, drinking and gardening. Three water sources in Buganzo, Buyagu and Kalangwa villages were observed (2 protected and 1 unprotected water source). The immediate surroundings were clean, not muddy, with no human feces, animal droppings or other waste around them. However, at one of the water sources, we observed livestock drinking from the source environs. In another, there were indications that people bathe at the water source.

Data suggest that 48.9% and 27.3% HHs had access to 15 or more litrrs of water within 30 min from a protected water source in wet and dry seasons, respectively (see Table 2). In the schools, all pupils fetched water for school use, including washing latrines, making meals and cleaning classroom floors. Water was carried from outside of the campus except for one school with a borehole in the compound. Most of the time pupils carried at least 5 L of water per day. None of the schools sampled provided boiled or chlorinated drinking water for pupils. Data suggest that all the girls fetched water while at school (five times a week; at least 5 L) and at home (once to thrice daily; at least 20 L per day). Girls and women fetch water at home, the boys and men rarely do. 

In one of the FGDs, the youth indicated that water sources were inadequate because most of the sources were still too far from the households. They claimed that water availability worsens during the dry season. This was affirmed by one of the women FGDs (the second) and the men’s second FGD which independently stated that they did not experience improvement in water access despite the intervention. The men claimed that there were no other water sources nearby except one shallow well shared by more than 400 households in the village. However, this was contradicted by the first women FGD and the first men’s FGD. The group suggested that as a result of the project intervention, water access was nearer to them. The differences in opinions could be attributed to the proximity of water locations to the sampled respondents. Our survey data suggested that respondents close to the water sources accessed water within a short time compared to those far from the water source. The approximate duration for fetching water ranged between 10 min to an hour depending on HH location. The KIIs, indicated that those who would spend more than two hours fetching water prior to the intervention, had reduced to an hour or less. Respondents in one FGD mainly made up of men lacked understanding on the difference between clean and unsafe drinking water. They also doubted the willingness of people in the community to boil water for drinking. They expressed a dislike for boiled water, claiming that it altered taste and was therefore not “sweet” (palatable). However, the youth and women FGDs contradicted the men’s FGD regarding boiling water. The women and youth FGDs stated that they boiled water for drinking. Our survey data suggest that 36% of the respondents attempted to make water safe for drinking using different approaches. Of these, only 8.8% of the HHs boiled it and 3.2% chlorinated the water to make it safe for drinking as shown in Table 2. 

### 3.5. Efficiency and Effectiveness of the Interventions

We examined the project management in relation to time, cost and quality of work outputs. The intervention was aid funded, made some foreign exchange gains against the weakening Tanzanian currency (the shilling). This enabled more money for boreholes to be available. The cost of sinking a borehole was 23 million TShs (11,500 USD) hydrological survey fees inclusive.

Six latrines (3 for boys and 3 for girls) and fourteen teacher pit latrines were constructed in three out of 18 schools. The project had planned for two teacher pit latrines in each school (i.e., 36 latrines in total). We elucidate the efficacy of the choice of activities in relation to the intervention objectives in the discussion section. Nonetheless, our survey data suggested that 281 (52.4% of the respondents received a message/information on sanitation and hygiene through community meetings 135 (47%). The most common sources of the message/information were village leaders 187 (65%) and government agents 55 (19.1%). 

### 3.6. Sustainability of the Project

The major repairs or routine maintenance of boreholes was likely to be a challenge due to limited technical skills and finance. However, there was a willingness to maintain the water sources if sufficient community members were trained. Most importantly, skills as well as constant technical, couching or mentoring support are critical to successful rural water, sanitation and hygiene interventions [27]. We found that 18 water user groups (WUGs) had been established and trained. However, the training was insufficient to solve mechanical problems associated with boreholes and the WUGs did not have the tools for the task. Conversely, the WUGs collect funds from households and earmark the funds for maintenance purposes. Households were required to pay 10,000 TShs. user fees per year. Nonetheless, some HHs did not pay this amount at all or regularly due to unaffordability. The community resolved that every member should pay the user fees but there was no clear course of action to recover money from defaulters. Remarkably, we found that there was gender awareness in the water source governance. Women were represented on the WUGs committees, but their participation in the decisions regarding the water source governance was not assessed. 

### 3.7. Attitudes and Behavior Change

We observed poor sanitation and hygiene practices in the surveyed communities, some HHs inappropriately disposed infants’ feces in gardens, rubbish pits, and in bushes. This was confirmed in two FGDs (1 men; 1 women). Another FGD highlighted that some HH disposed infant feces in latrines while others left them (feces) for the dogs to “cleanup” (eat). The inappropriate disposal of feces was rooted in a traditional belief that barred women from disposing of children’s feces in the pit latrines. 

“Those children whose faeces are disposed in pit latrines suffer retarded growth (the children do not achieve expected growth milestones)” Women FGD. 

One of the youth FGD asserted that women and men use latrines more than the children. However, another youth FGD stated that it was mostly women, because they are at home the longest. The latter group also stated that older men avoid being seen going to the latrines because their culture considers older men going to a latrine as shameful. When the need to defecate arises, men go to the bushes, but give excuses so as not to be embarrassed. They go with a hoe pretending to go gardening; this finding is consistent with our survey data on open defecation. Most people use plant leaves (majority), old or used paper, water (for a few minorities) and soil for anal cleaning after defecation. Hand washing after defecation was not a common practice; many respondents were unsure why they should be washing hands after defecation. Those who knew about the practice of hand washing said to have been taught during clinic visits or during village committee meetings. Respondents said more should be done to educate the communities on hand washing to change their beliefs. Our findings suggest that 281 (52.4%) HHs had received a message or materials on sanitation and hygiene for the four months recall period. The most common places where messages were received were at community meetings for 135 (47%) of respondents, government offices (53), at home (30), on radio (29), posters and/or leaflets in community (21) and at health centers (10). The main sources of the messages were the village leaders 187 (65%) and government agents 55 (19.1%).

## 4. Discussion

We examined a four-year water and sanitation intervention for the 35,978 inhabitants of a rural community; what worked and what did not work and why. We found that despite the interventions to increase water supply, improve sanitation and hygiene, the incidence of diarrhea increased. However, our data on diarrhea may not reflect the true situation due to the limitation on measuring and determining diarrhea, and differences in seasonality at the time of the study. Notwithstanding the data limitations, investing in the construction of latrines for teachers at school consumed many resources. The resources could have been dedicated to diarrhea interventions targeting children under five years old. Nonetheless, construction of latrines for teachers was relevant to the context. The latrines could have had positive outcomes in the education of children which we did not investigate as it was not the focus of our study. 

Diarrheal diseases related to inadequate water supply and sanitation are among the leading causes of death among children in the developing world [28,29,30]. Reversing the trends requires a critical examination of effective strategies in diarrheal related morbidity and mortality reduction [31]. Our data suggest that the interventions did not effectively tackle diarrhea morbidity. This could possibly be attributed to strong cultural ties that discourage appropriate excreta disposal. Other diarrhea morbidity correlates were: ineffective hygiene and sanitation promotion, poor choice of activities, and inadequate water quality interventions specifically at the point of use. Boiling or chlorinating water were not strongly emphasized or monitored during the intervention. Boiling or chlorinating water at the point of use, combined with increased sanitation and hygiene coverage effectively combat diarrhea morbidity and mortality [32,33,34]. Culturally competent activities such as the engagement of elders to break the strong rooted traditional beliefs that discourage appropriate excreta disposal were not implemented. This was a missed opportunity to leverage influential community systems and structures to boost participation for the desired change. Active participation in rural sanitation and hygiene interventions strongly correlates to improved sanitation and hygiene outcomes [7,8]. 

Factors responsible for the sustained incidence of diarrheal diseases were open defecation, inappropriate infant fecal disposal and temporary latrines that unusable during the wet season. Other factors included the absence of hand washing facilities after latrine use, poor knowledge on hand washing and clean and, safe drinking water. This could imply that the preferred interventions especially the education campaigns and their champions were ineffective. Some studies have alluded to the ineffectiveness of educational campaigns because of their prescriptiveness, they weaken home-grown solutions, target the easy to reach, and are not provocative enough to challenge the status quo [35,36]. The persistent practice of open defecation may have exacerbated the occurrence of diarrheal in the area [34]. In some places, the soil type (sandy) was unsuitable for digging and sustaining pit latrines while using the rudimentary technology. Notwithstanding the sandy soils, the cost of VIP latrines remains unaffordable by many households. However, the amount stated by the men’s FGD is comparable to the government estimate of USD 600 per latrine stance (USD 12,000 per latrine block of 20 stances) across the country [11]. Similarly, an earlier Dutch government report observed that the Dutch government funded projects in Shinyanga region abandoned VIP latrines due to high costs of construction [22]. Our study found that primary barriers to HH acquisition of latrines were cost and sandy soils which hampered digging standard pits. This could explain the persistent 40% open defecation captured in the survey. The combination of these factors may explain the escalation of diarrhea disease incidence. 

The pupil to latrine stance ratio (1:68 boys; 1:64 girls) in schools exceeded the Tanzanian government recommended ratio of 1:25 for boys and 1:20 for girls. Furthermore, the school based water and hygiene interventions missed out on targeting the under five year old children. This age group is too young to enroll but it is the most critical and sensitive to diarrheal diseases. Nonetheless, the intervention targeted school children on the assumption that the knowledge and skills acquired by the children would be replicated at home to influence hygiene [23].

The water supply positively impacted the HHs that were close to the new water sources in terms of time reduction and energy expended in fetching water. However, this was not quantified. The FGDs gave different responses to water access. Some FGDs claimed there was no difference in access before and after the intervention while others suggested they had better access. Nonetheless, our survey data suggest that HHs closer to water sources spent less time fetching water compared to HHs that were not in close proximity. This could explain the differences in opinions of the FGD respondents. In the KIIs, it was indicated that those who would spend more than two hours fetching water, had reduced to an hour or less. More than half of individuals surveyed accessed less than 15 L of water per day. This quantity fell below the average amount of 20 L per person per day recommended by WHO [28]. We suggest the community water interventions consider providing a modest supply as stipulated in the national standards. In this case, the Tanzanian government standard is 25 L of potable water per person per day [13]. Notwithstanding the water access, there were limited efforts to make the water safe for drinking. Evidence suggests that activities like boiling of water or applying chlorine bleach reduces the incidences of water borne diseases such as diarrhea [37,38]. By failing to emphasize simple water treatment as a core tenet, the intervention missed the opportunity to optimize the health outcomes. 

The WUGs’ active participation assured short term functionality of the water sources unless they enforce full compliance of user fees collection. The community resolved that every member should pay the user fees but there was no clear course of action to recover money from defaulters. The course of action on defaulters would be more effective if set by the community members in consideration of their contextual factors such as genuine unaffordability. Households unable to pay user fees could provide other services such as monitoring and/or manual labor required for cleaning and maintenance unless exempted by old age, chronic illness or being child headed. Even so, the burden of repairing broken boreholes remains high. The smallest village had 170 HHs and the largest had 889 HHs. Assuming 90% compliance on annual collections would amount to the equivalent of USD 765 to 4000 per annum. A borehole replacement required $12,300. Based on these estimates, the smallest village would take 16 years to accumulate enough money to replace a borehole; the largest village would require three years. The mean village size was 369 HHs, 90% compliance would generate USD1, 845 per annum. It would take seven years to replace a broken or non-discharging borehole in this theoretical model. Unless the communities can afford such costs, there is need to explore other avenues such as partnership with the local government or the private sector for intervention sustainability [39]. Sustaining ground water sources worried many respondents who stated that major repair jobs may not be managed by WUGs alone. We challenge the choice of technology used especially the boreholes which appear simple but remain complex to rural communities. They often break into disuse due to poor maintenance and poor understanding of the technology. The alternative is piped water, which could be cost effective and easy to maintain because pipes and taps are easily accessible for repair when required [6,40]. However, the long distance from the nearest pipe grid, the absence of feasibility study and/or the inability to engage with the relevant government department were obstacles that could have been addressed by the intervention. Even so, piped water also requires relevant skills for maintenance. 

Our findings suggest that 52.4% (281) of the respondents received a message on sanitation and hygiene to promote the desired positive behavioral outcomes. The rest of the households were not reached due to limited choice of strategies which mainly included home visits and community educational meetings. In order to reach all (100%), 5–6 households needed to be visited each working day. With the project full time staff of only two individuals, this was unlikely to be accomplished. In addition, it takes more than giving messages and talks to change behavior. A prior understanding of health seeking behavioral models coupled with establishing knowledge, attitudes and practices (KAP) at intervention inception stage is essential [41]. 

There was time lost before starting the intervention, due to procurement bottlenecks, especially borehole drilling and constructing VIP latrines in schools. This could have been avoided by preselection of suppliers at the conceptual stage of the intervention. Additionally, the quality of the water was tested before the commissioning of each borehole, but there were no periodic tests thereafter. Some boreholes failed for various reasons; one ran dry after a short period of operation, one had high iron content, and was rejected by the users and another was broken or vandalized. This reflects insufficient hydrological understanding prior to sinking of the boreholes and poor community ownership of the vandalized water source. Some risks were anticipated and well managed. For example, the drilling company was liable for hydrological survey outcomes during the drilling period to avoid dry boreholes. However this could have been strengthened by prolonging the period after drilling to avoid boreholes running dry after a short period. In addition, HH water storage and usage (including treatment at point of use) should have been considered. This omission could in part explain the diarrhea disease escalation. However, a formal investigation is necessary to identify the causal effect. We suggest that there was an insufficient monitoring mechanism to capture the increase in diarrhea and trigger a follow up action. 

### 4.1. Lessons Learnt 

In the intervention design phase, a knowledge, attitude and practice (KAP) survey was not included. A KAP survey would have informed the focus of the educational campaign for effective sanitation and hygiene outcomes. 

Improving access to water may have helped some HHs. Scaling up the intervention to cover the entire community including the hard to reach areas would have been more effective. Furthermore, matching water provision with improved sanitation and hygiene would have halted or reduced diarrheal incidence. Studies in Bangladesh, Kenya, Mozambique and Uganda have demonstrated that incidences of diarrheal diseases are not only a result of poor access to water, but how it is stored, used and excreta disposal, and hygiene [35,42,43,44]. For sustainability, the water user fees would be the major source of finance to repair or replace broken boreholes or to get new protected wells dug. More attention should have been put to achieving the highest possible level of compliance in paying the user fees. Moreover, having an integrated skilling strategy at the intervention inception would ensure the affordability of borehole maintenance and optimize their functionality using local technicians.

### 4.2. Specific Recommendations on How to Maximize Benefits for This Rural Community

Ground water supply to communities has challenges of seasonal variability, water quality and accessibility. We recommend that piped water should be tapped from the national grid or other alternative sources, especially for Nyamigege and Busangi villages, which are only 5–10 km away from the closest grid path. However, a feasibility study is necessary to establish the cost and compare it with the cost of other infrastructure such as sinking boreholes. Alternative rural water sources such as springs have been successfully harnessed and piped to communities using local energy sources other than electricity in Ethiopia [39,45]. Boreholes remain technologically challenging and costly to manage and maintain for the rural people. 

Rural water, hygiene and sanitation interventions should be tackled with a public health approach rather than general community development. This could be achieved by engaging qualified public health practitioners to guide the intervention and establishing appropriate health outcome monitoring mechanism at the conceptual stage. Generalist community development personnel lack the expertise to effectively implement and monitor such technical interventions. The intervention in question had two full time staff and four part-time staff to implement and monitor, however none of the staff had a public health background. The staff found the physical infrastructure (boreholes, wells and latrines) an attractive measure of success and accountability because it is visible and tangible. A public health specialist would focus beyond the physical infrastructure to monitor and demonstrate the overall health outcomes.

There is need for a comprehensive collaboration with the government and other stakeholders to share the burden of major infrastructure repairs and replacement of water sources. Alternatively, the affected communities should be encouraged to revise the user fee charge as appropriate and enforce compliance. 

Stakeholders could consider extending a small loan concept to WUGs for water and hygiene infrastructure maintenance similar to a successful NGO-BRAC model in Bangladesh [45]. This would provide access to appropriate sanitation and water infrastructure while earning interest for the WUGs and increasing their reserve funds for maintaining the water system. As the funds grow, the WUGs could invest into more water sources as well as more influence in sanitation, hygiene practices, and water treatment and storage. 

### 4.3. Limitations

There was no baseline survey conducted, most of the baseline data used were from secondary sources such as the intervention assessment report and health center records. There were no monitoring data to establish the number and frequency of educational campaigns conducted to establish their effectiveness and saturation. Some of the data were self-reported, subject to recall bias and, in some cases, respondents could have reported what they thought the interviewer wished to hear (courtesy bias). We did not carry out water quality testing at the water source using tests such as *E. coli* water contamination. Data on diarrhea may not reflect the true situation due to the limitation on measuring and determining diarrhea, and differences in seasonality at the time of the survey (since diarrhea follows a season pattern). Further longitudinal studies may be required to confirm findings observed in our study. The assessment of diarrhea occurrence was not active surveillance; the milder forms could have been missed. The study area population was not revised since the inception of the intervention. Perhaps there were more people coming into the area during the intervention period, causing a gain dilution factor. We were unable to revise the area population because of logistical and budgetary constraints. 

## 5. Conclusions

This study focused on the rural Busangi community that previously attracted dismal scholarly attention. We outlined practical lessons and highlighted the challenges faced by NGOs in providing community services. We evaluated an intervention that improved water supply and access in terms of the amount per person per household and the distance to water source. Nonetheless, the achievements did not prevent water contamination responsible for the incidence of diarrheal diseases in children under five years. The provision of water alone to rural communities is insufficient to address challenges associated with waterborne diseases. The intervention would have had better outcomes by emphasizing water supply alongside quality monitoring, consumer behavior, and sanitation conditions. Similar interventions would benefit from continuous use of standardized monitoring for contamination, sanitation, and hygiene behavior. Rural WATSAN interventions should continuously be adjusted based on emerging evidence. To wait for end of intervention evaluation in order to learn of the outcomes as often practiced by NGOs inadvertently undermines the purpose of the intervention and is an infringement of the users’ rights and dignity. 

## Figures and Tables

**Table 1:** Demographic Characteristics of HH respondents’ characteristics.

Characteristics	Number of Households
**Ward**	
Busangi	151
Chela	266
Ntobo	130
**Age**	Mean 39 years (SD 14)
	Median 36
**Level of education attained**	
Primary school	351 (65.9%)
Secondary School	51 (9.5%)
Tertiary	2 (0.4%)
Never attended school	129 (24.1%)
**Primary occupation**	
Subsistence farmer	462 (86.2%)
Self Employed	29 (5.4%)
Stay home	11 (2.1%)
Others	34 (6.3%)

**Table 2:** Summary of household survey data.

Characteristics	Percentage (2011) *	Number/Percentage (2015)
*Water borne diseases (Morbidity)*		
Had children with diarrhea in past 2 weeks	0.4%	52 (9.7%)
Bloody diarrhea	0.1%	11 (2.1%)
Watery diarrhea	7.1%	41 (7.7%)
Skin infections	na	15 (2.8%)
*Hand washing and drying racks*		
HH with hand washing facilities at the latrine	na	45 (8.4%)
HH with the tippy taps	na	26 (4.9%)
HH with drying racks for utensils	90.1%	339.1(63.9%)
*Water Access*		
Percent doing a round trip < 30 min in the wet season	19.2%	262 (48.9%)
Percent doing a round trip < 30 min in the dry season	17.6%	146 (27.3%)
Percent of HH with access to 15 L per person per day irrespective of source type	na	240 (44.8%)
*Point of use water treatment*		
HH that attempted to make water safe for drinking	na	195 (36%)
HH that boil their water	na	47 (8.8%)
HH that filter water with a cloth	na	71(13.3%)
Those that bleach with chlorine	na	17 (3.2%)
*Excreta disposal (Pit Latrine Use & non-use)*		
Number of HH with a pit latrine	na	346 (64.5%)
Number of HH with a VIP latrine	na	103 (19.2%)
Number of HH with a pit latrine not useable	7.6%	13 (2.4%)
Number that open defecate (OD) when in public places	na	212 (40%)
HH whose children OD in wet season	na	113 (21%)
HH whose children feces are buried or thrown into bushes	na	112 (20.5%)
*Knowledge of WASH*		
Agreed that owing a pit latrine:		
Improves hygiene	na	296 (55.2%)
Improves safety	na	62 (11.6%)
Improves health	na	263 (49.1%)
Gives more privacy	na	96 (17.9%)
Safe disposal of human waste maintains good hygiene		208 (39%)

* denotes secondary data from health center records and assessment report; na denotes not available.

**Table 3:** Water and sanitation situation in selected Busangi ADP schools, 2015.

School	Total # of Pupils	# of Boys	# of Girls	# of Teachers	# of Latrine Blocks **	Ratio to Girls	Ratio to Boys	Hand Washing Facilities	Waste Disposal Pits	Water Source ^†^
Buyagu	351	150	201	9	2	1:34	1:25	Absent	Present	one
Buchambaga	262	136	126	8	2	1:21	1:23	Absent	Absent	Absent
Gula	585	288	297	-	2	1:50	1:48	Absent	Absent	Absent
Kalagwa	354	170	184	7	2	1:31	1:28	Present	Absent	Absent
Buganzo	331	165	166	28	2	1:28	1:28	Present	Present	Absent
Busangi	790	383	407	-	2	1:64	1:68	Present	Present	Absent

** One latrine block has six rooms (stances) each; boys and girls use separate latrine blocks; ^†^ denotes water source, e.g., borehole within the school perimeter; **#** denotes number.
